# Seven key actions to eradicate rheumatic heart disease in Africa: the Addis Ababa communiqué

**DOI:** 10.5830/CVJA-2015-090

**Published:** 2016

**Authors:** David Watkins, Liesl Zuhlke, Mark Engel, Rezeen Daniels, Veronica Francis, Gasnat Shaboodien, Bongani M Mayosi, Mabvuto Kango, Azza Abul-Fadl, Abiodun Adeoye, Sulafa Ali, Mohammed Al-Kebsi, Fidelia Bode-Thomas, Gene Bukhman, Albertino Damasceno, Dejuma Yadeta Goshu, Alaa Elghamrawy, Bernard Gitura, Abraham Haileamlak, Abraha Hailu, Christopher Hugo-Hamman, Steve Justus, Ganesan Karthikeyan, Neil Kennedy, Peter Lwabi, Yoseph Mamo, Pindile Mntla, Chris Sutton, Ana Olga Mocumbi, Charles Mondo, Agnes Mtaja, John Musuku, Joseph Mucumbitsi, Louis Murango, George Nel, Stephen Ogendo, Elijah Ogola, Dike Ojji, Taiwo Olabisi Olunuga, Mekia Mohammed Redi, Kamanzi Emmanuel Rusingiza, Mahmoud Sani, Sahar Sheta, Steven Shongwe, Joris van Dam, Habib Gamra, Jonathan Carapetis, Diana Lennon

**Affiliations:** Department of Medicine, Groote Schuur Hospital and University of Cape Town, South Africa; University of Washington, USA; Department of Medicine, Groote Schuur Hospital and University of Cape Town, South Africa; Department of Medicine, Groote Schuur Hospital and University of Cape Town, South Africa; Department of Medicine, Groote Schuur Hospital and University of Cape Town, South Africa; Department of Medicine, Groote Schuur Hospital and University of Cape Town, South Africa; Department of Medicine, Groote Schuur Hospital and University of Cape Town, South Africa; Department of Medicine, Groote Schuur Hospital and University of Cape Town, South Africa; PAS CAR Secretariat, South Africa; African Union Commission, Ethiopia; Association Friends of Children with RHD, Egypt; University College Hospital, Ibadan, Nigeria; University of Khartoum and Sudan Heart Centre, Sudan; University of Sana’a, Yemen; University of Jos, Nigeria; Harvard Medical School/ Partners in Health, USA; Mozambican Heart Association (AMOCOR), Mozambique; Addis Ababa University, Ethiopia; Ministry of Health, Rheumatic Heart Disease Programme, NCDs, Egypt; Kenyatta National Hospital, Kenya; Jimma University, Ethiopia; Mekelle University, Ethiopia; Ministry of Health and Social Services, Namibia; Touch Foundation, Tanzania; All India Institute of Medical Sciences, India; College of Medicine, University of Malawi, Malawi; Uganda Heart Institute, Uganda; Technical Adviser, NCD directorate, Federal Ministry of Health of Ethiopia, Ethiopia; Sefako Makgatho Health Sciences University, South Africa; Sefako Makgatho Health Sciences University, South Africa; Instituto Nacional de Saúde, Mozambique and PAS CAR Vice President South; Mulago Hospital, Uganda; University Teaching Hospital, Zambia; University Teaching Hospital, Zambia; Rwanda Heart Foundation, Rwanda; East African Community, Burundi; PAS CAR Secretariat, South Africa; University of Nairobi, Kenya; University of Nairobi, Kenya; University of Abuja Teaching Hospital, Nigeria; Federal Medical Centre, Abeokuta, Nigeria; Common Market for Eastern and Southern Africa, Ethiopia; University of Rwanda, Rwanda; Bayero University Kano and Aminu Kano Teaching Hospital, Nigeria; Cairo University Children Hospital, Faculty of Medicine, Egypt; WHO Regional Office in Africa, Congo; Novartis, USA; African Heart Network, Tunisia; Telethon Kids Institute, University of Western Australia, Princess Margaret Hospital for Children, Australia; University of Auckland, New Zealand

**Keywords:** rheumatic heart disease, prevention

## Abstract

Acute rheumatic fever (ARF) and rheumatic heart disease (RHD) remain major causes of heart failure, stroke and death among African women and children, despite being preventable and imminently treatable. From 21 to 22 February 2015, the Social Cluster of the Africa Union Commission (AUC) hosted a consultation with RHD experts convened by the Pan-African Society of Cardiology (PASCAR) in Addis Ababa, Ethiopia, to develop a ‘roadmap’ of key actions that need to be taken by governments to eliminate ARF and eradicate RHD in Africa.

Seven priority areas for action were adopted: (1) create prospective disease registers at sentinel sites in affected countries to measure disease burden and track progress towards the reduction of mortality by 25% by the year 2025, (2) ensure an adequate supply of high-quality benzathine penicillin for the primary and secondary prevention of ARF/RHD, (3) improve access to reproductive health services for women with RHD and other non-communicable diseases (NCD), (4) decentralise technical expertise and technology for diagnosing and managing ARF and RHD (including ultrasound of the heart), (5) establish national and regional centres of excellence for essential cardiac surgery for the treatment of affected patients and training of cardiovascular practitioners of the future, (6) initiate national multi-sectoral RHD programmes within NCD control programmes of affected countries, and (7) foster international partnerships with multinational organsations for resource mobilisation, monitoring and evaluation of the programme to end RHD in Africa.

This Addis Ababa communiqué has since been endorsed by African Union heads of state, and plans are underway to implement the roadmap in order to end ARF and RHD in Africa in our lifetime.

## Abstract

While acute rheumatic fever (ARF) essentially vanished from industrialised countries during the latter half of the 20th century,^1^ the condition and its major sequel, rheumatic heart disease (RHD) remain important public health concerns in Africa. Poverty and inadequate primary healthcare systems are major contributors to the persistence of ARF/RHD in Africa.^2^ On the other hand, improving economic conditions and enhanced health system investments during the HIV/AIDS era offer an opportunity to address this neglected disease of poverty in a co-ordinated fashion.^3^,^4^

Over the past decade, there has been a renewed global interest in RHD as well as a proliferation of scientific and public health work led by African investigators and practitioners.^5^ At the same time, the World Heart Federation (WHF) non-communicable disease action plan, developed for the World Health Assembly in 2013, called for a 25% reduction in premature mortality from RHD by the year 2025 (‘25 by 25’).^6^

Prior to 2015, two workshops on ARF/RHD in Africa were held, with resultant position statements on the necessary steps to address ARF/RHD on the continent. The first statement, the ‘Drakensberg Declaration on the Control of Rheumatic Fever and Rheumatic Heart Disease in Africa’, was issued in 2005 after the meeting in South Africa,^7^ and the second, the ‘Mosio- tunya Call to Action’, was issued in 2014 after the meeting in Zambia.^8^ This was followed by the publication of a key dataset, enumerating key characteristics, gaps in implementation of evidence-based practices and shortfalls in the management of RHD in African communities.^9^

From 21 to 22 February 2015, the Social Cluster of the African Union Commission (AUC) hosted the Third All-Africa Workshop on ARF and RHD, which was an expert consultation of RHD clinicians and researchers affiliated with the Pan-African Society of Cardiology (PASCAR). This meeting was intended to develop a roadmap that could be adopted by ministries of health and governments in order to eliminate ARF and control RHD in their home countries. This article outlines the Addis Ababa communiqué that emerged from the consultative meeting, and also provides a brief report of the objectives and proceedings of the meeting, as well as the outcomes of the meeting in the first six months thereafter.

## The Addis Ababa communiqué on eradication of RHD in Africa

## Motivation

The communiqué began by recalling that RHD is both preventable and common in Africa, affecting 1.5 to 3% of school-aged children.^10^,^11^ Because severe RHD is lethal in the absence of surgical treatment,^12^ the total economic cost of premature mortality in Africa is staggering,^13^ and hampers the achievement of the Millennium Development Goals and forthcoming Sustainable Development Goals on health. The problem has been made worse by a lack of comprehensive, integrated prevention and control programmes in most African Union (AU) member states that carry a heavy burden of ARF/RHD.

The AU recognised several mandates to convene this meeting and discuss a roadmap for ARF/RHD in Africa. These included the following:

The 6th ordinary session of the Conference of AU Ministers of Health (CAMH6; 22–26 April 2013), adopted under the AU Executive Council Declaration EX.CL/Dec.795(XXIV): this requested the AU commission (AUC) to develop a mechanism to control NCDs in Africa.
The first joint AU and World Health Organisation (WHO) ministerial meeting, convened under AU Assembly Decision Assembly/AU/Dec.506(XXII): this pledged action towards controlling NCDs in Africa under the AUC–WHO joint work plan (14–17 April 2014).
The Drakensberg Declaration and the Mosi-o-Tunya Call to Action, mentioned above, which were endorsed by the WHO Regional Office for Africa and called for the eradication of ARF/RHD ‘in our lifetime’.

## Barriers to action

The foundation of the recommendations of the communiqué was a recent publication of baseline characteristics of patients with RHD from 12 African countries.^9^ Several of the key barriers to control of RHD in Africa are listed in Table 1. Notably, despite the lack of progress on RHD control in Africa, there are several examples of countries, such as Cuba,^14^ Costa Rica,^15^ and Tunisia,^16^ that have realised the eradication of ARF and control of RHD over several years by implementing co-ordinated and comprehensive public health programmes.

**Table 1:** Barriers to ARF/RHD eradication in Africa

1. Lack of RHD surveillance efforts at the local, regional and national level.
2. Variable supply and use of high-quality benzathine penicillin G.
3. Low use of reproductive health services among women with RHD.
4. Overly centralised diagnostic and treatment services for RHD.
5. Few facilities capable of providing cardiac surgery for advanced RHD.
6. Lack of national RHD prevention programmes.
7. Absence of multi-sectoral RHD initiatives.

## Meeting objectives and proceedings

The objectives of the Third All-Africa Workshop on ARF and RHD were as follows:

develop approaches on how to eradicate RHD in Africa
develop milestones for the eradication of RHD
identify key stakeholders for collaboration in eradication of RHD.

The meeting was officially opened on 21 February by His Excellency the AU Commissioner for Social Affairs, Dr Mustapha Kaloko. Opening comments were provided by representatives from the Government of the Federal Democratic Republic of Ethiopia, the African Union Commission Department of Social Affairs, the WHO Regional Office in Africa, and Novartis/ Sandoz Pharmaceuticals.

Three main activities comprised the meeting:
Formal presentations on successful ARF/RHD control programmes in Africa and Oceania.
Breakout sessions on the minimal datasets that are needed, key investments that will be required, and stakeholder participation that should be sought in order to develop ARF/RHD programmes in African countries.
A group deliberation on the final set of expert recommendations and key principles to be enumerated in the communiqué.

At the close of the meeting, the principles of the communiqué, reproduced in Table 2., were assented to, and the document was sent by the AUC to the April 2015 Ministerial Conference on Health, Population and Drug Control for consideration.

**Table 2:** The Third All-Africa Workshop on ARF and RHD: recommendations to the AU commission and member states

1. Establish prospective RHD registers at sentinel sites in affected member states in order to monitor RHD-related health outcomes, including the achievement of a 25% reduction in mortality from RHD by the year 2025.
2. Ensure adequate supplies of high-quality benzathine penicillin that can be administered in the most effective manner, in order to achieve primary and secondary prevention of RHD.
3. Guarantee universal access to reproductive health services for women with RHD and other NCDs, in whom pregnancy carries specific and often fatal risks, and for whom contraception can reduce maternal and foetal mortality.
4. Decentralise appropriate technical expertise to the primary and district levels in order to improve the diagnosis of ARF (which is under-diagnosed in Africa) and early detection, diagnosis, secondary prevention and treatment of RHD using cross-cutting point-of-care technologies such as cardiac ultrasound, anticoagulation testing and rapid antigen tests for group A streptococcal pharyngitis.
5. Establish centres of excellence for cardiac surgery, which will sustainably deliver state-of-the-art surgical care, train the next generation of African cardiac practitioners, and conduct research on endemic cardiovascular diseases, including RHD.
6. Foster multi-sectoral and integrated national RHD control programmes led by the Ministry of Health, which will oversee the implementation of national RHD action plans in order to achieve the goal of reducing mortality from RHD and other NCDs by 25% by the year 2015.
7. Cultivate, through a strong communication framework, partnerships between the AUC, ministries responsible for health, international agencies, governments, industry, academia, civil society and other relevant stakeholders, in order to ensure the implementation of the above actions, and the connection of African RHD control measures with the emerging global movement towards RHD control.

## Recommendations

1. Establish prospective RHD registers. These would occur at sentinel sites in AU member states affected by ARF/RHD. The major objective of these registers will be to monitor progress towards RHD-related health outcomes, which include a 25% reduction in premature mortality from RHD by the year 2025.
2. Ensure adequate supplies of benzathine penicillin G (BPG). The WHO recognises BPG as an essential medication. In order to achieve adequate coverage of primary and secondary prevention measures for ARF/RHD, BPG must be readily available at all primary care facilities in AU member states, and training of providers on effective and safe use of BPG should be part of supply-side efforts. BPG can also be used for the treatment of other endemic diseases in Africa, such as syphilis, yaws and sickle cell disease.
3. Guarantee universal access to reproductive health services for women with RHD. RHD greatly increases a woman’s risk of mortality and foetal demise during pregnancy. Reproductive health services, including contraception, are currently underutilised among women with RHD in Africa and this contributes to the high maternal mortality rates on the continent. Comprehensive care for RHD and other NCDs should include access to reproductive health services for all women at risk.
4. Decentralise diagnostic services for ARF/RHD to district hospitals. Primary healthcare services and district hospitals need appropriate technical expertise in the diagnosis of ARF and RHD. Key point-of-care technologies that should be considered for provision at district and community levels include ultrasound of the heart (echocardiography), anticoagulation testing, and antigen tests for the rapid diagnosis of group A streptococcal pharyngitis.
5. Establish cardiac surgery centres of excellence. Such facilities could sustainably deliver state-of-the-art surgical care as well as train the next generation of African cardiac specialists. They could also be centres of research on endemic cardiovascular diseases (including RHD).
6. Foster multi-sectoral and integrated national RHD control programmes led by ministries responsible for health. These programmes would oversee the implementation of national RHD action plans and progress towards the ‘25-by-25’ targets.
7. Cultivate partnerships that can implement the actions above. A partnership needs to be developed between the AU commission, ministries responsible for health, international agencies, governments, industry, academia, civil society and other relevant stakeholder to monitor and evaluate progress related to the implementation of the key actions and achievement of the outcome of 25% reduction in premature mortality from RHD by the year 2025.

In addition to these recommendations, important and specific roles for international stakeholders Table 3. were identified. Finally, the communiqué requested the AU to mandate PASCAR and other stakeholders to work with the AU commission to develop a detailed implementation plan of the key actions. This would include roles and responsibilities, timelines, estimates of costs, and a communication framework for the roadmap.

**Table 3:** The Third All-Africa Workshop on ARF and RHD: recommendations to the AU commission and member states

1. Provide open-access resources to develop and strengthen ARF/RHD country programmes.
2. Raise the profile of RHD in the context of strengthening and equity of health systems.
3. Partner with AU member states to address the supply of high-quality benzathine penicillin G.
4. Support development of an ARF vaccine that would be affordable and effective in Africa.

## Adoption and next steps

On 14 April 2015, the Addis Ababa communiqué was presented to the African Union Specialised Technical Committee on Health, Population and Drug Control (a platform of ministers of health, population and drug control) where it was adopted unanimously and referred for further consideration to the African Union Heads and Government Summit.^17^ In their meeting held from 7 to 12 June 2015 in Johannesburg, South Africa, the 27th ordinary session of the Executive Council (Ministers of Foreign Affairs) adopted the document under declaration number EX.CL/Dec.876(XXVII), and it was endorsed by the 25th AU Heads of State and Government Summit that was held from 14 to 15 June 2015 in Johannesburg, South Africa.

Following on the formal adoption of the principles of the expert consultation, it is now incumbent on ministries of health of AU member states to develop local implementation plans. The PASCAR ARF/RHD task force is developing principles for implementation of ARF/RHD action plans at the local level, which will include an analysis of the key financial and human resource investments required in order to accomplish the objectives of the Addis Ababa communiqué. To this end, PASCAR and the AUC plan to convene a workshop in March 2016 on the implementation of the Addis Ababa communiqué at country level.

## Conclusions

Over the past 25 years, ARF and RHD have endured on the African continent despite dramatic progress in the control of many other important childhood, adolescent and maternal conditions.^18^,^19^,^20^ The partnership between the African Union Commission and the Pan-African Society of Cardiology, and the subsequent political commitment to the principles for the eradication of ARF/RHD in Africa, promises to change this situation. It is hoped that the implementation of action plans on ARF/RHD will, through a concerted and multi-sectoral effort, rapidly improve cardiovascular health and strengthen health systems for chronic non-communicable diseases in Africa.

## References

[1] Gordis L (1985). The virtual disappearance of rheumatic fever in the United States: lessons in the rise and fall of disease. T. Duckett Jones memorial lecture.. Circulation.

[2] Robertson KA, Mayosi BM (2008). Rheumatic heart disease: social and economic dimensions.. S Afr MedJ.

[3] Watkins DA, Zuhlke LJ, Engel ME, Mayosi BM (2009). Rheumatic fever: neglected again.. Science.

[4] Robertson KA, Volmink JA, Mayosi BM (2006). Towards a uniform plan for the control of rheumatic fever and rheumatic heart disease in Africa – the Awareness Surveillance Advocacy Prevention (A.S.A.P.) Programme.. S Afr Med J.

[5] Maurice J (2013). Rheumatic heart disease back in the limelight. Lancet.

[6] Remenyi B, Carapetis J, Wyber R, Taubert K, Mayosi BM (2013). World Heart Federation. Position statement of the World Heart Federation on the prevention and control of rheumatic heart disease.. Nature Rev Cardiol.

[7] Mayosi B, Robertson K, Volmink J (2006). The Drakensberg declaration on the control of rheumatic fever and rheumatic heart disease in Africa.. S Afr Med J.

[8] Mayosi BM, Gamra H, Dangou J-M, Kasonde J (2014). Rheumatic heart disease in Africa: the Mosi-o-Tunya call to action.. Lancet Global Health.

[9] Zuhlke L, Engel ME, Karthikeyan G (2015). Characteristics, complications, and gaps in evidence-based interventions in rheumatic heart disease: the Global Rheumatic Heart Disease Registry (the REMEDY study).. Eur Heart J.

[10] Carapetis JR, Steer AC, Mulholland EK, Weber M (2005). The global burden of group A streptococcal diseases. Lancet Infect Dis.

[11] Marijon E, Mirabel M, Celermajer DS, Jouven X (2012). Rheumatic heart disease.. Lancet.

[12] Gunther G, Asmera J, Parry E (2006). Death from rheumatic heart disease in rural Ethiopia.. Lancet.

[13] Watkins D, Daskalakis A (2015). The economic impact of rheumatic heart disease in developing countries.. Lancet Global Health.

[14] Nordet P, Lopez R, Duenas A, Sarmiento L (2008). Prevention and control of rheumatic fever and rheumatic heart disease: the Cuban experience (1986–1996–2002).. Cardiovasc J Afr.

[15] Arguedas A, Mohs E (1992). Prevention of rheumatic fever in Costa Rica. J Pediatr.

[16] Ben Romdhane H, Haouala H, Belhani A (2005). [Epidemiological transition and health impact of cardiovascular disease in Tunisia]. La Tunisie Medicale.

[17] African Union

[18] Wang H, Liddell CA, Coates MM (2014). Global, regional, and national levels of neonatal, infant, and under-5 mortality during 1990-2013: a systematic analysis for the Global Burden of Disease Study 2013.. Lancet.

[19] Kassebaum NJ, Bertozzi-Villa A, Coggeshall MS (2014). Global, regional, and national levels and causes of maternal mortality during 1990–2013: a systematic analysis for the Global Burden of Disease Study 2013.. Lancet.

[20] Murray CJ, Ortblad KF, Guinovart C (2014). Global, regional, and national incidence and mortality for HIV, tuberculosis, and malaria during 1990–2013: a systematic analysis for the Global Burden of Disease Study 2013.. Lancet.

